# 4-Hydroxy-2-nonenal induces apoptosis by activating ERK1/2 signaling and depleting intracellular glutathione in intestinal epithelial cells

**DOI:** 10.1038/srep32929

**Published:** 2016-09-13

**Authors:** Yun Ji, Zhaolai Dai, Guoyao Wu, Zhenlong Wu

**Affiliations:** 1 State key Laboratory of Animal Nutrition, College of Animal Science and Technology, China Agricultural University, Beijing, 100193, P. R. China; 2 Department of Animal Science, Texas A&M University, College Station, TX 77843, USA

## Abstract

Excessive reactive oxygen species (ROS) induces oxidative damage to cellular constituents, ultimately leading to induction of apoptotic cell death and the pathogenesis of various diseases. The molecular mechanisms for the action of ROS in intestinal diseases remain poorly defined. Here, we reported that 4-hydroxy-2-nonenal (4-HNE) treatment led to capses-3-dependent apoptosis accompanied by increased intracellular ROS level and reduced glutathione concentration in intestinal epithelial cells. These effects of 4-HNE were markedly abolished by the antioxidant L-cysteine derivative N-acetylcysteine (NAC). Further studies demonstrated that the protective effect of NAC was associated with restoration of intracellular redox state by Nrf2-related regulation of expression of genes involved in intracellular glutathione (GSH) biosynthesis and inactivation of 4-HNE-induced phosphorylation of extracellular signal-regulated protein kinases (ERK1/2). The 4-HNE-induced ERK1/2 activation was mediated by repressing mitogen-activated protein kinase phosphatase-1 (MKP-1), a negative regulator of ERK1/2, through a proteasome-dependent degradation mechanism. Importantly, either overexpression of MKP-1 or NAC treatment blocked 4-HNE-induced MKP-1 degradation, thereby protecting cell from apoptosis. These novel findings provide new insights into a functional role of MKP-1 in oxidative stress-induced cell death by regulating ERK1/2 MAP kinase in intestinal epithelial cells.

Excessive generation of reactive oxygen species (ROS) and/or defective antioxidant activity contributes to cellular redox imbalance, which is a critical pathogenic factor associated with various diseases^1^,^2^. Intestinal epithelium is constantly exposed to reactive oxygen metabolites from luminal contents or systemic oxidants which are rapidly removed by anti-oxidant systems, and a defect in this pathway leads to reversible or irreversible cellular injury^3^. Persistently elevated ROS triggers genetic or epigenetic alterations, resulting in oxidative damage to cell constituents (e.g. proteins, lipids, and nucleic acids), ultimately leading to induction of apoptotic cell death and the pathogenesis of various gastrointestinal disorders including peptic ulcers, gastrointestinal cancers, and inflammatory bowel disease^1^,^4^,^5^. In addition, several lines of studies show that enteral commensal or probiotic bacteria in the lumen of small intestine affect diverse homeostatic functions, including regulation of cellular growth, maintenance of barrier function, and modulation of immune responses by targeting the intestinal redox-oxidant balance^6^,^7^, suggesting a critical role of redox in intestinal epithelial survival and homeostasis^3^.

4-Hydoxy-2-nonenal (4-HNE) is originally identified as an end product formed by the reaction of ROS with polyunsaturated fatty acids during oxidative stress^8^,^9^. Growing evidence indicates that 4-HNE can function as an important second messenger and, therefore, has been implicated in the regulation of various cellular processes, including cell proliferation, differentiation, apoptosis, inflammatory response and endoplasmic reticulum stress^8^,^10^,^11^,^12^. A number of signaling proteins involved in cell proliferation or apoptotic cell death signaling pathways, such as p53^13^, protein kinase B (also known as AKT)^14^,^15^, and mitogen-activated protein (MAP) kinases^16^,^17^ are regulated by 4-HNE and contribute to cell proliferation or cell death in multiple types of cells. As one of the main cell types that constitute the intestinal barrier, intestinal epithelium forms a single layer and separates the intestinal luminal contents from the internal environment, ensuring the absorption of nutrients and irons and also preventing the passage of harmful or unwanted substances from entering the circulation. The proper function of the intestinal barrier is maintained by the well-controlled balance between cell proliferation and apoptosis in which ROS may play a regulatory role^18^. First, intestinal epithelium has a highly metabolic rate with a rapid turnover within 3–4 days, compared with other organs, which confers to the ROS generation in the milieus^19^. Second, intestinal epithelial cells are constantly exposed to antigens, toxins, and commensal or pathogenic bacteria, which can activate cellular defense or detoxification system accompanied by elevated ROS production^19^,^20^. All these findings indicate that the intestine is highly susceptible to the damaging effect of ROS and its metabolite, including 4-HNE.

It has been reported that 4-HNE induces apoptotic cell death by regulating the expression of proteins involved in cell death signaling pathways^21^, as well as proteins implicated in stressor (such as H_2_O_2_, UV, heat, and oxidant chemicals) -triggered apoptosis^12^. Despite the new knowledge of 4-HNE in the regulation of various cellular processes, the cellular response to 4-HNE and underlying apoptotic mechanisms in normal intestinal epithelium remains unknown. In this study, we reported that incubation of intestinal epithelial cells with 4-HNE led to caspase 3-dependent apoptosis, which was abolished by the antioxidant L-cysteine derivative, N-acetylcysteine (NAC). The protective effect of NAC was associated with restoration of redox state and inactivation of 4-HNE-induced extracellular signal-regulated protein kinases ERK1/2 phosphorylation through repressing mitogen-activated protein kinase phosphatase-1 (MKP-1).

## Results

### NAC attenuated 4-HNE-induced cell death in intestinal epithelial cells

**Figure 1:**
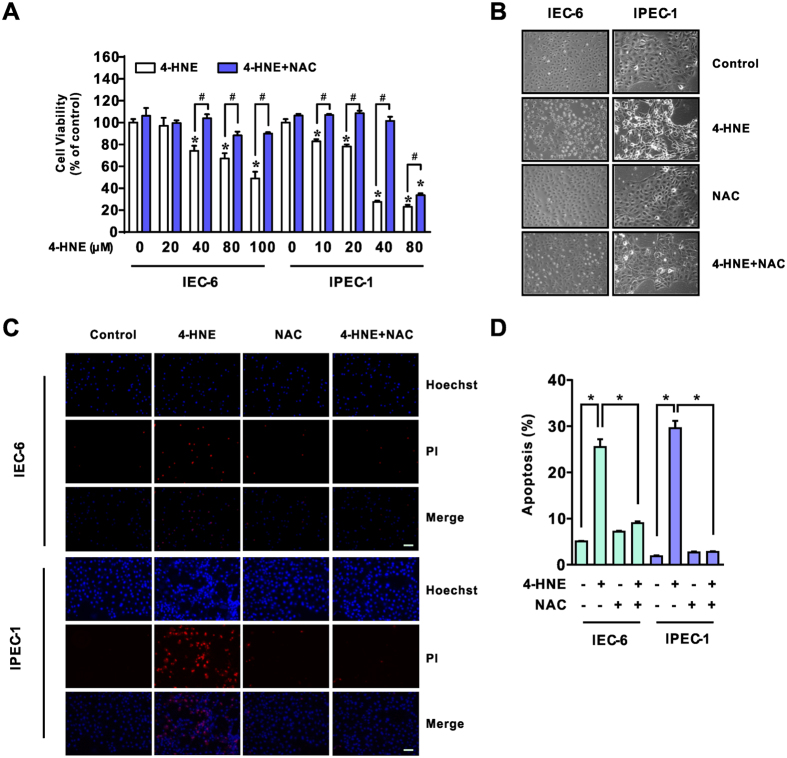
NAC attenuated 4-HNE-induced cell death in intestinal epithelial cells. (**A**) Cells were pre-incubated in serum-free medium with or without NAC (5 mM) for 2 h and then were exposed to various concentrations of 4-HNE as indicated for an additional 8 h. Cell viability was evaluated using the cell counting kit-8 (CCK-8) reagent, n = 6. *p < 0.01 compared with the control, ^#^*p* < 0.01 compared with 4-HNE alone. (**B**) Morphological observations of IEC-6 and IPEC-1 cells exposed to 4-HNE (80 μM for IEC-6 and 40 μM for IPEC-1, respectively) in the presence or absence of NAC (5 mM) for pretreatment. (**C**) Hoechst 33342 and PI staining of cells. Cells were treated as in Fig. 1B and then were stained with Hoechst 33342 (5 μg/ml) and PI (2.5 μg/ml), magnification x100. (**D**) Statistical analysis for Hoechst 33342/PI double positive cells treated as in Fig. 1C. Cell numbers were counted from three independent micrographs for each treatment, *p < 0.01.

To assess the cytotoxic effect of 4-HNE and a potential role of antioxidant on 4-HNE- induced oxidative stress, normal small intestinal epithelial cells (IEC-6 and IPEC-1) pretreated with or without NAC (5 mM, 2 h) were exposed to 4-HNE for indicated time points. As shown in Fig. 1A, 4-HNE treatment led to decreased viability of both IEC-6 and IPEC-1 cells in a dose-dependent manner (Fig. 1A). However, the reduced cell viability observed upon 4-HNE treatment was markedly attenuated by NAC treatment in both cell lines (Fig. 1A). Morphological observation using phase contrast microscopy demonstrated that 4-HNE treatment resulted in a significant increase of floating cells, appearance of cell shrinkage and boundary contraction as compared with control cells in both cell lines, which were markedly reversed by NAC treatment (Fig. 1B). This morphological alteration was confirmed by Hoechst 33342 and propidium iodide (PI) staining (Fig. 1C,D), suggesting a protective effect of NAC on 4-HNE induced cell death in intestinal epithelial cells.

### The protective effect of NAC on 4-HNE-induced cell death was associated with inactivation of caspase 3-dependent apoptosis

**Figure 2:**
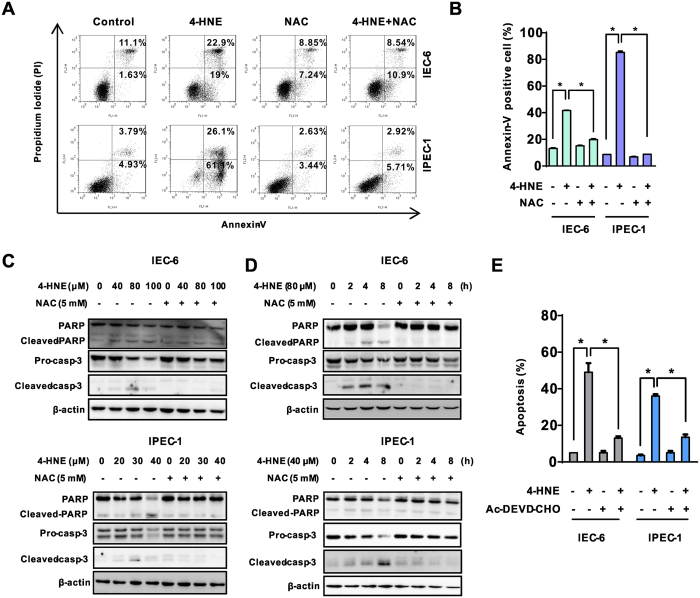
The protective effect of NAC on 4-HNE-induced cell death was associated with inactivation of caspase-3-dependent apoptosis. (**A**) Cells pretreated with or without NAC (5 mM) in serum-free medium for 2 h and then were exposed to 4-HNE (80 μM for IEC-6 and 40 μM for IPEC-1, respectively) for an additional 8 h. Cell apoptosis was evaluated using the Annexin-V/PI double staining kit by flow cytometry. (**B**) Statistical analysis for Annexin-V positive cells treated as in Fig. 2A. Values are expressed as the mean ± SEM (n = 3), *p < 0.01. (**C**) Immunoblotting analysis of pro-caspase-3, cleaved caspase-3 and PARP in cells treated with indicated concentrations of 4-HNE for 8 h in the presence or absence of NAC pretreatment (5 mM). (**D**) Immunoblotting analysis for pro-caspase-3, cleaved caspase-3 and PARP in cells treated with 4-HNE (80 μM for IEC-6 and 40 μM for IPEC-1, respectively) with or without NAC pretreatment for indicated time periods; β-actin was used as a loading control. (**E**) Caspase-3 inhibitor Ac-DEVD-CHO protected cells from 4-HNE-induced apoptosis. Cells pretreated with Ac-DEVD-CHO (50 μM) for 1 h were treated with 4-HNE (80 μM for IEC-6 and 40 μM for IPEC-1, respectively) for 8 h and and cell apoptosis was determined by Hoechst 33342/PI double staining. Values are expressed as the mean ± SEM (n = 3), *p < 0.01.

To investigate the characteristic of cell death as observed, NAC-pretreated cells were incubated with or without 4-HNE and then were collected for apoptosis assays, including FACS and Western blot analysis. As shown, 4-HNE single treatment resulted in marked cell death as indicated by an increased number of apoptotic cells (Fig. 2A,B) which were mostly abolished by the NAC. In agreement with observed phenotype, 4-HNE treatment resulted in the accumulation of cleaved caspase-3 and PARP, two characteristics of apoptosis, in both the dose- (Fig. 2C) and time-dependent manner (Fig. 2D). Notably, the effects of 4-HNE were largely abolished by NAC (Fig. 2C,D). In contrast, NAC single treatment did not induce apoptosis as evidenced by FACS analysis and Western blot analysis in both cell lines. Importantly, inhibition of caspase 3 by Ac-DEVD-CHO protected cells from 4-HNE-induced apoptosis (Fig. 2E). These results indicated that 4-HNE exposure triggered caspase 3-dependent apoptosis in intestinal epithelial cells which can be abrogated by an antioxidant NAC.

### Nrf2-related restoration of intracellular redox states contributed to the protective effect of NAC on 4-HNE-induced apoptosis

**Figure 3:**
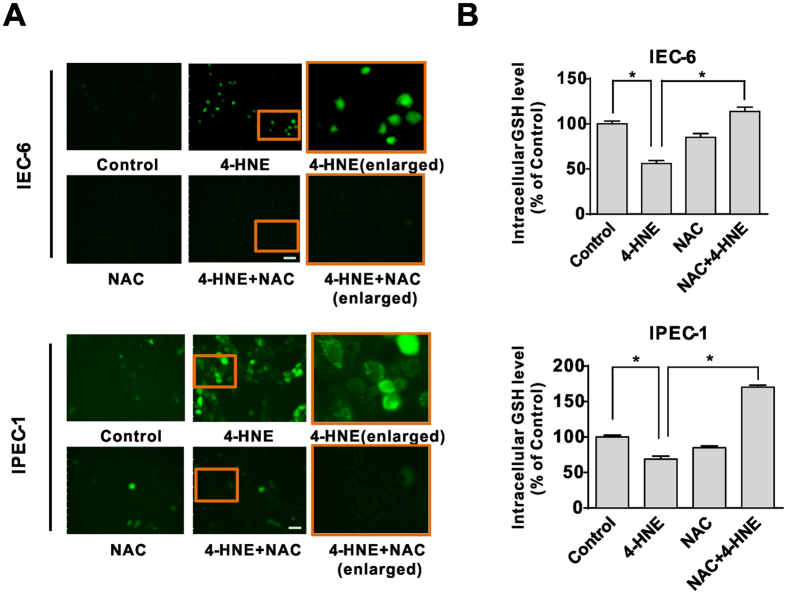
The restoration of intracellular redox states contributed to protective effect of NAC on 4-HNE-induced apoptosis. (**A**) ROS production triggered by 4-HNE was blocked by NAC pretreatment. Cells pretreated with or without NAC (5 mM, 2 h) in serum-free medium were loaded with the DCFH-DA probe (10 μM) and then were treated 4-HNE (80 μM for IEC-6 and 40 μM for IPEC-1) for 2 h, the fluorescence was measured immediately using a fluorescence microscope. (magnification x100) (**B**) The intracellular GSH levels in epithelial cells. Cells pretreated with or without NAC (5 mM, 2 h) were treated with 4-HNE (80 μM for IEC-6 and 40 μM for IPEC-1) for 6 h. The intracellular GSH levels were determined by high performance liquid chromatography (HPLC). Values are expressed as the mean ± SEM (n = 3), *p < 0.01.

**Figure 4:**
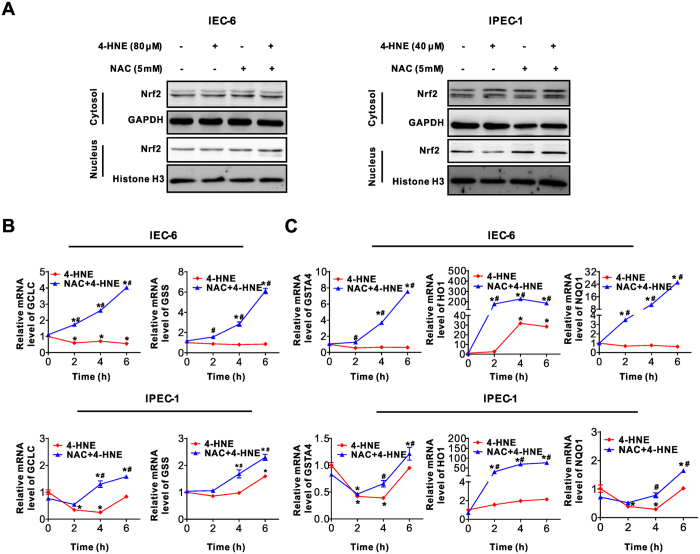
Nrf2-related signaling was activated by NAC pretreatment in 4-HNE-treated cells. (**A**) Cytoplasmic and nucleus protein levels for Nrf2. Cells pre-treated with or without NAC (5 mM) for 2 h were treated with 4-HNE for 2 h. Cytoplasmic and nuclear fractions were extracted and the protein levels for Nrf2 were analyzed by Western blot. GAPDH and Histone H3 were used as loading controls for the fraction of cytoplasmic or nucleus protein, respectively. (**B**) mRNA levels for GCLC and GSS in intestinal epithelial cells. Cells pre-treated with or without NAC (5 mM, 2 h) were treated with 4-HNE (80 μM for IEC-6 and 40 μM for IPEC-1) for indicated time points, qRT-PCR was performed to determine the mRNA expression level for GCLC and GSS. β-actin was used as the reference control. Values are expressed as the mean ± SEM (n  =  3), *p < 0.01 compared with that of 0 h control of the same treatment, ^#^*p* < 0.01 compared with that of 4-HNE treatment without NAC pre-incubation at the same time points. (**C**) mRNA levels for HO1, NQO1 and GSTA4 in intestinal epithelial cells. Cells were treated as in Fig. 3C and qRT-PCR was performed to determine the mRNA levels for indicated genes, β-actin was used as the reference control. Values are expressed as the mean ± SEM (n = 3), *p < 0.01 compare with that of 0 h control of the same treatment, ^#^*p* < 0.01 compared with that of 4-HNE exposure without NAC pretreatment at the same time point.

As NAC is a potent antioxidant^22^, we next explored the intracellular ROS production in intestinal epithelial cells treated with 4-HNE, NAC, or NAC and 4-HNE co-treatment. As demonstrated, 4-HNE treatment led to increased ROS levels as evidenced by the DCFH-DA assay, which were significantly alleviated by NAC (Fig. 3A). Similarly, the concentration of intracellular GSH, the main redox molecule, decreased following 4-HNE treatment, which was markedly reversed by NAC in both IEC-6 and IPEC-1 cells (Fig. 3B). Considering that the nuclear factor erythroid 2–related factor 2 (Nrf2) is a critical regulator for cellular response to antioxidant defense^23^, we next examined the effect of 4-HNE, NAC, or 4-HNE + NAC on nucleus and cytoplasmic content of Nrf2 protein. As showed, 4-HNE or NAC treatment has modest effect on Nrf2 (Fig. 4A) in both cell lines. In contrast, 4-HNE and NAC co-treatment increased Nrf2 protein in nuclear fractions (Fig. 4A). Consistently, the mRNA expression of GCLC, the catalytic subunit of GCL (glutamate-cysteine ligase, also known as γ-glutamylcysteine synthetase (γ-GCS), and glutathione synthetase (GSS), two essential enzymes for the synthesis of glutathione^24^,^25^) were significantly upregulated by 4-HNE and NAC combination treatment at 4–6 h post-treatment compared with that of 4-HNE single treatment in both IEC-6 and IPEC-1 cells (Fig. 4B). Additionally, the mRNA levels for Nrf2 downstream targets, including HO1 (heme oxygenase 1), NQO1 (NAD(P)H:quinone oxidoreductase 1), and GSTA4 (glutathione S-transferase alpha 4) (Fig. 4C) were markedly up-regulated in 4-HNE and NAC co-treated cells compared with cells treated with 4-HNE alone. All these data indicated that 4-HNE-induced cell apoptosis was associated with GSH depletion and downregulation of Nrf2 related antioxidant redox signaling. Accordingly, exogenous NAC abolished 4-HNE-induced cell death by an Nrf2-related increase in expression of genes involved in GSH biosynthesis and antioxidant response in intestinal epithelial cells.

### Modulating of ERK1/2 activation was responsible for the apoptotic effect induced by 4-HNE

**Figure 5:**
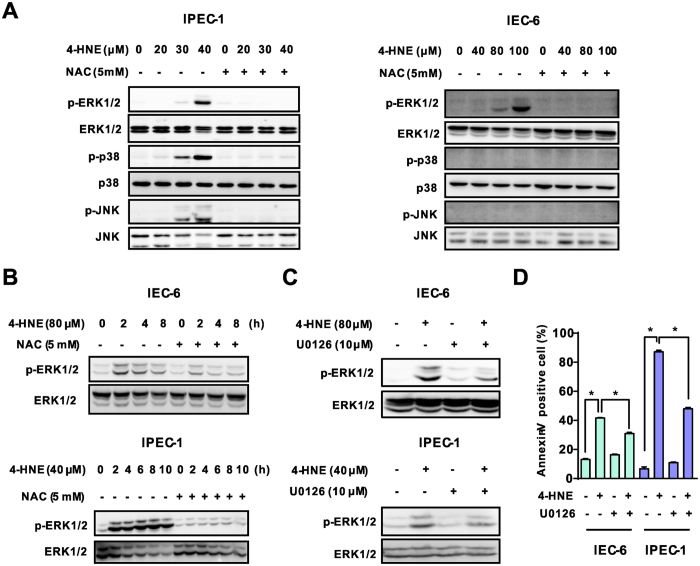
Modulating of ERK1/2 activation was responsible for the apoptotic effect induced by 4-HNE. (**A**) Western blot analysis of MAP kinase expression in intestinal epithelial cells. Cells pretreated with NAC (5 mM, 2 h) were exposed to 4-HNE (80 μM for IEC-6 and 40 μM for IPEC-1) for 8 h, the protein levels of ERK1/2, p-ERK1/2, p38 MAPK, p-p38 MAPK, JNK and p-JNK were determined using indicated antibodies. β-actin was used as the loading control. (**B**) Western blot analysis of ERK1/2 activation in a time dependent manner. Cells pretreated with NAC (5 mM, 2 h) were exposed to 4-HNE for different time periods. The protein levels of ERK1/2, and p-ERK1/2 were determined. β-actin was used as the loading control. (**C**) Western blot analysis of ERK1/2 activation in intestinal epithelial cells treated with 4-HNE in the presence or absence of U0126. Cells pretreated with U0126 (10 μM, 1 h) were treated with indicated concentrations of 4-HNE for 2 h. The protein levels of ERK1/2, and p-ERK1/2 were determined. β-actin was used as the loading control. (**D**) Inhibition of ERK1/2 activation blocked 4-HNE-induced cell apoptosis. Cells were treated with 4-HNE (80 μM for IEC-6 and 40 μM for IPEC-1) for 8h after U0126 pretreatment (10 μM, 1 h), and then cell apoptosis was determined by Facs analysis. Values are expressed as the mean ± SEM (n = 3), *p < 0.01.

Mitogen-activated protein kinases (MAPK), including extracellular signal–regulated kinase1/2 (ERK1/2), c-Jun N-terminal kinase/stress-activated protein kinase (JNK/SAPK), and p38 MAP kinase are well known kinases involved in the regulation of apoptosis and cell survival in response to various stresses, including ROS^26^,^27^. We next determined protein expression profiles using Western blot in both IEC-6 and IPEC-1 cells treated with 4-HNE, NAC or 4-HNE in combination with NAC treatment. 4-HNE exposure led to elevated levels of protein phosphorylation, including ERK1/2, JNK and p38 MAP kinases in IPEC-1 cells which are mostly attenuated by NAC (Fig. 5A). In contrast, 4-HNE treatment resulted in activation of ERK1/2 without affecting the phosphorylation of JNK and p38 MAP kinases in IEC-6 cells (Fig. 5B). The inactivation of ERK1/2 phosphorylation by NAC in 4-HNE-treated cells were further confirmed in the time course experiment involving both IEC-6 and IPEC-1 cells (Fig. 5B), suggesting that ERK1/2 activation might be responsible for the cell death effects caused by 4-HNE in intestinal epithelial cells. To further demonstrated the functional role of ERK1/2 activation in 4-HNE-induced apoptotic effects, cells pretreated with or without U0126, a specific inhibitor of the ERK1/2^28^, were incubated in the presence or absence of 4-HNE. Our results indicated that ERK1/2 inhibitor U0126 abrogated 4-HNE-induced ERK1/2 phosphorylation (Fig. 5C) and cell death (Fig. 5D) in both IEC-6 and IPEC-1 cells. In contrast, 4-HNE-induced cell death was not rescued by SB203580, a p38 inhibitor, or SP600125, a JNK inhibitor in IPEC-1 cells (Data not shown). These data indicated that ERK1/2, instead of p38 MAPK or JNK, is responsible for 4-HNE-mediated apoptosis in intestinal epithelial cells. Antioxidant NAC prevented 4-HNE-induced ERK1/2 activation, thereby exerting an anti-apoptotic effect.

### PERK-eIF2α signaling was not involved in 4-HNE-induced ERK1/2 activation

**Figure 6:**
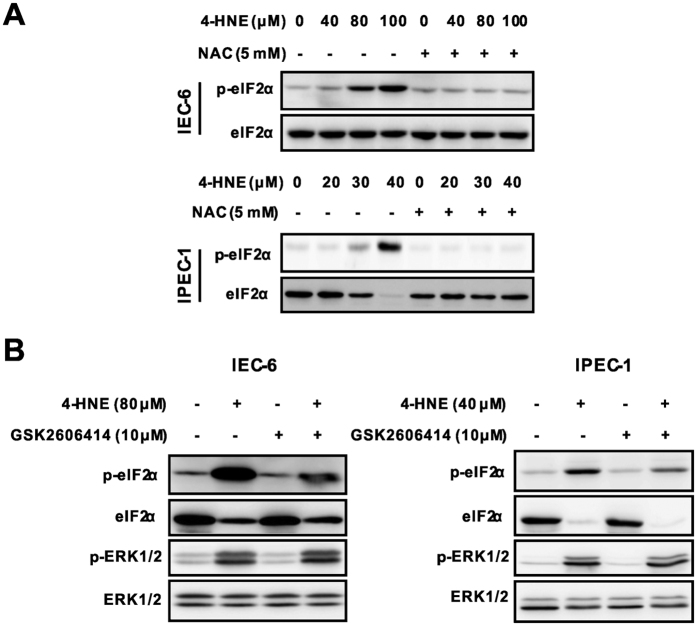
PERK-eIF2α signaling was not involved in 4-HNE induced ERK1/2 activation. (**A**) 4-HNE-induced eIF2α activation was inhibited by NAC pretreatment. Cells pretreated with NAC (5 mM, 2 h) were exposed to 4-HNE as indicated for 2 h, the protein levels of eIF2α and p-eIF2α were determined using indicated antibodies. β-actin was used as the loading control. (**B**) PERK inhibitor had no effect on 4-HNE-induced ERK1/2 activation. Cells pretreated with or without the GSK2606414 (10 μM, 1 h) were treated with 4-HNE as indicated for additional 1 h. The protein levels of ERK1/2 and p-ERK1/2 were determined in IEC-6 (left panel) and IPEC-1 cells (right panel).

P53 plays an important role for cell fate decision in response to oxidative stress^29^. Activation of p53 has been reported to contribute to 4-HNE-induced cell death by inducing downstream pro-apoptotic proteins including MAP kinases^13^. However, 4-HNE treatment failed to activate p53 and downstream target such as Bax and BCL-XL/S (Supplementary Figure S1A), and appeared not to be a regulator of ERK1/2 activation in our study. Recent study shows that ERK1/2 can be activated by PERK-eIF2α signaling in response to ROS and involved in caspase-dependent apoptosis^30^. As expected, 4-HNE treatment led to a markedly increase in the phosphorylation of eIF2α, which was largely abolished by NAC in both IEC-6 and IPEC-1 cells (Fig. 6A). To determine a potential regulatory relationship between eIF2α and ERK1/2, epithelial cells pretreated with a PERK inhibitor GSK2606414^31^, were exposed to 4-HNE. Western blot analysis revealed that PERK inhibitor GSK2606414 blocked 4-HNE-induced eIF2α activation, but had no effect on the protein level of ERK1/2 (Fig. 6B). Collectively, these results indicated that 4-HNE enhanced PERK-eIF2α signaling as previously reported^32^, but this signaling did not contribute to ERK1/2 activation in intestinal epithelial cells.

### MKP-1 was a critical regulator modulating ERK1/2 activation in response to oxidant exposure

**Figure 7:**
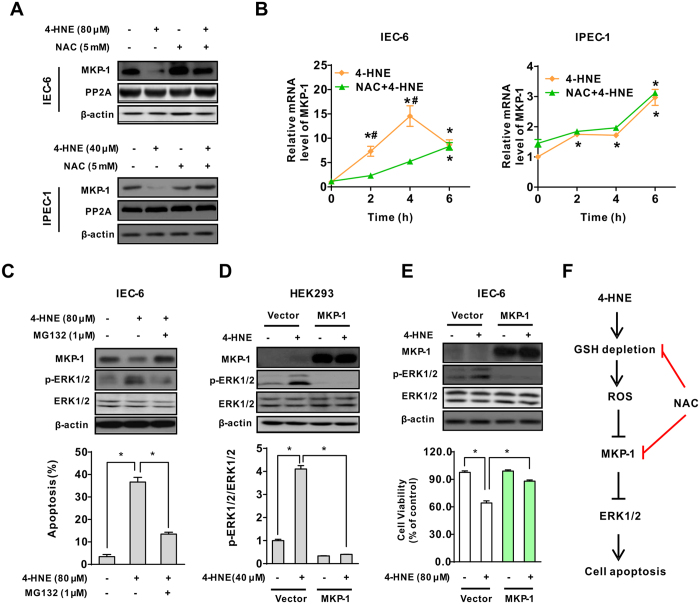
MKP-1 was a critical regulator modulating ERK1/2 activation following exposure to oxidant. (**A**) NAC prevented 4-HNE-induced MKP-1 down-regulation. Cells pretreated with NAC (5 mM, 2 h) were exposed to 4-HNE, the protein levels of MKP-1 and PP2A were determined using indicated anti-bodies. β-actin was used as the loading control. (**B**) Relative mRNA levels of MKP-1 in intestinal epithelial cells. IEC-6 (left panel) or IPEC-1 (right panel) cells pretreated with or without NAC were treated with 4-HNE, qRT-PCR was performed to determine the mRNA levels of MKP-1. β-actin was used as the reference control. Values are mean ± SEM (n = 3), *p < 0.01 compare with that of 0 h control of the same treatment. 4-HNE treatment control, ^#^*p* < 0.01 compared with that of 4-HNE alone at the same time point. (**C**) Protein degradation inhibitor blocked 4-HNE-induced MKP-1 down-regulation, ERK1/2 phosphorylation and apoptosis. IEC-6 cells were left untreated or treated with 4-HNE in the presence or absence of a proteolysis inhibitor MG132 (1 μM). The protein levels of MKP-1, ERK1/2, and p-ERK1/2 were determined. β-actin was used as the loading control. Cell apoptosis was evaluated by Hoechst 33342/PI staining. Values are expressed as mean ± SEM (n = 3), *p < 0.01. (**D**) MKP-1 repressed 4-HNE-induced ERK1/2 activation. HEK293 cells transfected with empty vector or MKP-1 vector were treated with 4-HNE for 2 h. The protein levels of MKP-1, ERK1/2, and p-ERK1/2 were determined. β-actin was used as the loading control. (**E**) Overexpression of MKP-1 abolished 4-HNE-induced ERK1/2 activation and cell death. IEC-6 cells transfected with empty vector or MKP-1 vector were treated with 4-HNE. The protein level of MKP-1, ERK1/2, and p-ERK1/2 (upper panel) and cell viability (lower panel) were determined. Values are mean ± SEM, *p < 0.01. (**E**) Proposed mechanism responsible for 4-HNE-induced apoptosis in intestinal epithelial cells.

As the activity of ERK1/2 is tightly regulated by the coordinated action of several protein phosphatases, we next investigated the possible involvement of protein phosphatases that responsible for the activation of ERK1/2 in intestinal epithelial cells. As shown, 4-HNE treatment led to down-regulation of the protein level of MKP-1, a main phosphatase that inactivates MAP kinases, without affecting the protein level of PP2A, in both IEC-6 and IPEC-1 cells (Fig. 7A). Importantly, NAC incubation reversed 4-HNE-induced decrease in MKP-1 protein level, indicating a potentially regulatory relationship between MKP-1 and ERK1/2 activation in epithelial cells. To explore whether this effect of 4-HNE on MKP-1 occurred at the transcriptional level, epithelial cells were treated with either 4-HNE or 4-HNE and NAC at different time points. Real-time PCR results showed that 4-HNE treatment led to a markedly increase in MKP-1 mRNA levels as earlier as 2 h post-treatment in both IEC-6 and IPEC-1 cells, and the values were further enhanced by NAC (Fig. 7B), thus excluding the possibility of regulation at the transcriptional level. Of interest, ERK1/2 phosphorylation and the MKP-1 down-regulation following 4-HNE treatment were reversed by a proteasome inhibitor MG132 (Fig. 7C, upper panel). Consistent with the changes in protein levels, 4-HNE-induced cell death was significantly abolished by MG132 (Fig. 7C, lower panel), indicating a post-translational regulation of MKP-1 in 4-HNE treated cells. To validate the repressing effect of MKP-1 on ERK1/2 activation, HEK293 cells transfected with empty vector or wild type MKP-1 vector were subjected to 4-HNE or left untreated. Overexpression of wild type MKP-1 significantly inhibited ERK1/2 phosphorylation in the presence of 4-HNE, and did not affect the abundance of total ERK1/2 protein (Fig. 7D). To confirm the anti-apoptotic effects of MKP-1 in epithelial cells, IEC-6 cells stably expressing wild type MKP-1 were selected and subjected to 4-HNE treatment. The introduction of wild type MKP-1 significantly attenuated 4-HNE-induced ERK1/2 activation and cytotoxicity (Fig. 7E). These results strongly suggested that repressing MKP-1 was critical for 4-HNE-induced ERK1/2 activation and cell death in intestinal epithelial cells. It is noteworthy that NAC abrogated 4-HNE-induced ERK1/2 activation by inhibiting MKP-1 degradation, thus improving cell survival in response to 4-HNE treatment (Fig. 7F).

## Discussion

ROS has been implicated in the pathogenesis of a variety of intestinal disorders, such as peptic ulcers, gastrointestinal cancers, and inflammatory bowel disease, due to its high metabolic rate and constantly exposure to luminal contents (e.g., toxin, commensal bacteria, and other factors) that contribute to ROS generation within the gastrointestinal tract^2^,^5^. Although aberrant regulation of redox homeostasis and oxidative stress in cancer cells has been well-studied, their roles in normal intestinal epithelium and gastrointestinal diseases remain unknown. Therefore, understanding the signaling pathways of ROS-related cell death would provide important clues to the mechanisms responsible for intestinal epithelial cell injury and the development of intestinal disease.

In the present study, we showed that 4-HNE, a major end product of lipid peroxidation and a widely accepted inducer of oxidative stress^9^,^33^, induced ROS- related cell death in both IEC-6 and IPEC-1, two widely used intestinal epithelial cells, as revealed by double staining assays, Facs, and immunoblot analysis. Of note, apoptosis of these cells was markedly reduced by NAC, a potent antioxidant. Although ROS has been reported to induce cell cycle arrest in several types of mammalian cell^34^, little is known about this effect in intestinal epithelial cells. However, both the cell cycle profiles and the protein levels of p21 and p27 (Data not shown), two inhibitory CDK regulators associated with cell cycle arrest, were not affected by 4-HNE treatment. Importantly, the pro-apoptotic effect of 4-HNE was associated with intracellular GSH depletion and activation of extracellular signal-regulated protein kinases ERK1/2 activation. Blockage of ERK1/2 activation by pharmacological inhibitor abrogated 4-HNE induced cell death, implicating ERK1/2 as a critical factor that responsible for the cell death effect (Fig. 7F). Accumulating data points to an essential role for ROS in the activation of autophagy^35^. However, the protein level of LC3, a marker of autophagy, was not affected by 4-HNE, NAC, or 4-HNE and NAC co-treatment (Supplementation Fig S1B), thus excluding the involvement of autophagic cell death triggered by 4-HNE in our study.

GSH is essential for cell survival as depletion of intracellular GSH results in massive apoptotic cell death in multiple cell lines^36^ or knock-out mice^37^. Conversely, overexpression of γ-GCS, a rate-limiting enzyme for GSH synthesis, has been shown to protect against apoptosis induced by stimuli that activate cell death pathways^38^. Despite much progress on 4-HNE-triggered apoptosis in cancer cells, there is very limited data in normal intestinal epithelial cells, especially considering that 4-HNE can accumulate up to concentrations of 10 μmol/L to 5 mmol/L^9^,^33^. In the present study, we found that 4-HNE exposure led to decreased GSH level and altered cellular redox state which is in agreement with previous studies^9^.

Nrf2 has also been shown to protect against oxidative stress-induced cell death by regulating enzymes involved in GSH biosynthesis as well as genes implicated in antioxidative defense^39^,^40^,^41^,^42^. Induction of antioxidant enzymes by activation of Nrf2 signaling has been considered as a promising strategy to combat with oxidative stress-related diseases^23^. In our study, we found 4-HNE treatment did not activate Nrf2 which is not in agreement with previous studies showing accumulation of nuclear Nrf2 upon oxidative stress^43^,^44^. One of the reasons for this discrepancy might due to a higher concentration of 4-HNE was used in our study compared with previous ones. In our pilot study, we found 2–5 μM 4-HNE induced cell proliferation (data not shown), in contrast, a higher concentration of 4-HNE induced cell death as shown herein and its underlying mechanisms. It has been also reported that low concentrations of 4-HNE induces adaptive response by activating Nrf2 signaling, while higher concentrations of 4-HNE impair Nrf2 nuclear accumulation and result in apoptosis^45^. Interestingly, NAC markedly up-regulated the mRNA level of enzymes (GCLC and GSS) involved in GSH synthesis, boosted Nrf2-related detoxifying capability by transcriptionally up-regulating expression of HO-1 and NQO1, phase 2 enzymes that participates in the scavenging of ROS^46^,^47^, and promoted the production of GSTA4 to remove intracellular 4-HNE (Fig. 4), thus restoring the redox and protecting intestinal epithelial cells from 4-HNE induce cell damage as previously reported^48^,^49^.

Mitogen-activated protein kinases (MAPKs), including the c-JunNH2-terminal kinases (JNK1⁄2), the p38 MAP kinases, and extracellular signal-regulated kinase (ERK1/2) signaling pathways, are well known to be activated in response to the intracellular redox state and oxidative stress, and potentially contribute to influencing cell survival or cell death^50^,^51^. To evaluate the functional role of MAP kinase signallings responsible for 4-HNE-induced apoptosis, we determined the activation of MAP kinases by Western blot analysis. As shown, ERK1/2, instead of p38 MAP kinase or JNK1/2, phosphorylation was markedly induced by 4-HNE in both IEC-6 and IPEC-1 cells, indicating a potential involvement of ERK1/2 in response to ROS insults in intestinal epithelial cells. Important, 4-HNE-induced ERK1/2 activation and cell death was markedly abolished by ERK1/2 inhibitor U0126 (Fig. 5), suggesting a critical role for ERK1/2 activation on cell death. This result is in agreement with data from renal epithelial cells^52^, alveolar epithelium^53^, or human proximal tubular epithelial cells^54^. Although the p53^13^ and PERK-eIF2α pathways^30^ have been reported to regulate ERK1/2 activation in response to various stresses, including 4-HNE, and contribute to cell death, this was not the observed in intestinal epithelial cells. In contrast, we found that activation of ERK1/2 following 4-HNE treatment was mediated by repressing MKP-1, a negative regulator of MAP kinases^55^. Such a regulatory relationship was further supported by ectopic expression experiments in both HEK293 and IEC-6 cells. Importantly, cells stably expressed wild type MKP-1 was resistant to 4-HNE induced ERK1/2 activation and cell death as compared with control cells (Fig. 7), indicating an anti-apoptotic effect of MKP-1 in response to oxidative stress. Additionally, our results also indicated that ERK1/2 was a preferred substrate repressed by MKP-1. First, ERK1/2 was markedly induced by 4-HNE which was accompanied by MKP-1 degradation in both IEC-6 and IPEC-1 cells (Fig. 7A), whereas the activation of p38 and JNK was modestly induced in IPEC-1 cells. Second, protein degradation inhibitor MG132 reversed ERK1/2 activation by abolishing MKP-1 down-regulation. Third, ectopic expression of wild type MKP-1 blocked 4-HNE-induced ERK1/2 phosphorylation and cell death but did not affect the protein levels of phosphorylated JNK and p38 MAPK (data not shown). Further studies are required to elucidate the underlying mechanisms responsible for this substrate preference.

In conclusion, 4-HNE exposure resulted in a caspase-3-dependent apoptosis in intestinal epithelial cells. This effect was associated with decreased intracellular GSH levels and activation of ERK1/2, which can be reversed by a potent antioxidant NAC. Mechanistically, the activation of ERK1/2 was mediated by proteasome-dependent protein degradation of MKP-1, a negative regulator of ERK1/2. NAC treatment restored intracellular redox states by Nrf2-related up-regulation of enzymes involved in GSH biosynthesis and genes involved in anti-oxidative defense, as well as inhibition of 4-HNE-induced MKP-1 degradation, thereby protecting cells from 4-HNE-induced apoptosis. The results from this study established a novel pathway that involves superoxide production, MKP-1 degradation and ERK1/2 activation in response to oxidative stress. Our results also support a role of NAC as a potential powerful antioxidant for prevention or therapy of intestinal disease caused by oxidative stress.

## Materials and Methods

### Reagents

4-Hydoxy-2-nonenal was bought from Cayman Chemical Co. (Ann Arbor, MI). N-Acetylcysteine was purchased from Sigma Chemical Co. (St. Louis, MO). DMEM/F12, DMEM and fetal bovine serum (FBS) were obtained from GIBCO BRL (Grand Island, NY). DCFH-DA was purchased from Beyotime Institute of Biotechnology (Haimen, China). Antibodies against PARP, MKP-1, Nrf2, p53, Bax, Bcl-xL/S, GAPDH, histone H3 and β-actin were obtained from Santa Cruz Biotechnology (Santa Cruz, CA). Antibodies against caspase-3, cleaved-caspase-3, total and phospho (p)-ERK1/2 (Thr202/Tyr204), JNK, p-JNK, p38 MAPK, p-p38 MAPK, eIF2α, p-eIF2α (Ser51), LC3A/B and PP2A were products of Cell Signaling Technology (Beverly, MA). Peroxidase-conjugated goat anti-rabbit and goat anti-mouse secondary antibodies were purchased from Huaxingbio Biotechnology Co. (Beijing, China). The annexin V-FITC&PI kit was from Jiamay Biotechnology (Beijing, China). Kinases inhibitors, including ERK1/2 inhibitor U0126, JNK inhibitor SP600125, p38 MAPK inhibitor SB202190, and protein degradation inhibitor MG132 were obtained from Cell Signaling Technology (Beverly, MA). PERK inhibitor GSK2606414 was from Selleck Chemicals (Houston, TX). Other reagents used in this study were ordered from Sigma, unless otherwise stated.

### Cell culture and drug treatment

The IEC-6 cell is a non-transformed rat small intestinal epithelial cell line, whereas the IPEC-1 cell is a stable epithelial cell line prepared from the jejunum of the new born pig. The IPEC-1 cells were grown in DMEM-F12 medium supplemented with 10% FBS and 1% penicillin–streptomycin (Gibco, NY). The IEC-6 cells were obtained from ATCC and cultured in DMEM medium containing 10% FBS and 1% penicillin–streptomycin. Both cell lines were incubated at 37 °C in a humidified chamber with 5% CO_2_. For drug treatment, cells pretreated with 5 mM NAC for 2 h were incubated with 4-HNE. Thereafter, cells were harvested for analysis. In some experiments, cells were pretreated with kinase inhibitors and then were exposed to be treated with 4-HNE.

### Cell viability assay

Cells seeded in 96-well plates at a density of 10,000 cells per well were treated as indicated in figures, and cell viability was determined by the using of Cell counting kit (Zoman Biotech, Beijing) according to the protocol provided by the manufacturer. After the treatment, 10 μL of CCK-8 was added to each well and then were incubated for 1 h. The optical density was determined at 450 nm using a Molecular Devices microplate reader.

### Apoptotic cell death assay

Cells were treated as indicated and apoptotic cell death was determined by Hoechst 33342/PI staining and flow cytometric analysis. For Hoechst 33342/PI staining, cells were harvested and stained with Hoechst 33342 (5 μg/mL) and propidium iodide (PI, 2.5 μg/mL) for 10 min. Then the stained cells were observed under fluorescence microscope (Zeiss, Germany). The morphology was also examined and images were captured under a light microscope connected with a digital camera (Nikon, Japan). For Flow cytometry analysis, harvested cells were stained with annexin V-FITC and PI for 15 min at room temperature. Flow cytometric analysis was performed on a FACSCalibur flow cytometer (BD Biosciences), and data were analyzed using the CellQuest software. The percentage of apoptotic cells was calculated as the percentage of the number of apoptotic cells over the total number of the cells.

### Determination of ROS production

The intracellular ROS production was determined by using DCFH-DA (2′,7′ dichlorofluorescein diacetate), an ROS-sensitive probe as previously described^56^. The DCFH-DA is a cell permeable indicator for ROS that is non-fluorescent until the acetate groups are removed and oxidation occurred in the cell and is irreversible converted into the fluorescent form DCF. For ROS measurements, cells were treated as indicated and DCFH-DA was introduced at a final concentration of 10 μmol/L and incubated at 37 °C for another 20 min. The intracellular ROS level was determined by a fluorescence microscope (Zeiss Axiowert).

### Measurements for reduced and oxidized glutathione

The reduced (GSH) and oxidized glutathione (GSSG) were determined by high-performance liquid chromatography (HPLC) as described^57^,^58^. In brief, cell samples were disrupted in homogenization buffer [12 mM iodoacetic acid:1.5 M HClO_4_ = 1:1(v/v)] by ultrasonic wave and centrifuged to deplete protein after 2 M K_2_CO_3_ was added. The S-carboxymethyl-glutathione, which forms a highly fluorescent derivative in the present of ortho-phthalaldehyde (OPA), was formed by using iodoacetic acid. The glutathione was analyzed by HPLC. HPLC separation was achieved in a C_18_ column (4.6 × 100 mm) with sodium acetate/methanol as solvents. Fluorescence at 480 nm was monitored after excitation at 394 nm.

### Quantitative real-time RT-PCR

Total RNA was extracted from cells using the Trizol reagent (CWBIO biotech Co., Beijing). Reverse transcription PCR was performed using the PrimeScript RT Reagent Kit (TaKaRa, Dalian, China), as instructed by the manufacturer and cDNA was used as template in the subsequent reactions. Real-time PCR was performed using SYBR Premix Ex Taq II (TaKaRa) and the ABI-Prism 7500 Sequence Detection System (Applied Biosystems) according to the instruction from the manufacturer. The primer sets used in this study were listed in supplementary Table S1. The mRNA level of β-actin was used as the internal control. The 2^−ΔΔCT^ method was used to determine the fold changes in mRNA levels of each sample, as compared with the reference sample.

### Immunoblot assays

Cells were harvested and lyszed on ice for 30 min in RIPA lysis buffer containing 50 mM Tris-HCl (pH 7.4), 150 mM NaCl, 1% NP-40, 0.1% SDS, 1.0 mmol/L PMSF, 1.0 mmol/L Na_3_VO_4_, 1.0 mmol/L NaF and protease inhibitor tablet (Roche, Indianapolis, IN), followed by sonication for 3 times with 10 s/time. The whole-cell lysates were centrifuged at 12,000 rpm for 10 min to collect the supernatants. The protein extraction from cytoplasmic and nucleus fractions of cells was conducted by using a kit from Beyotime Institute of Biotechnology (Haimen, China) following the instruction. The protein concentration of the supernatant was determined using the Pierce BCA protein Assay Kit (Huaxingbio, Beijing) with bovine serum albumin as standard. Equal amounts of protein (25 μg) were separated on SDS-page gels. Proteins were transferred to PVDF membranes (Millipore, Billerica, MA). The membranes were blocked in 5% fat-free milk in TBST (Tris-buffered saline with Tween 20) for 1 hour at room temperature, and then were incubated with indicated primary antibodies overnight at 4 °C. After incubation with horseradish peroxidase (HRP)-conjugated secondary antibody for 1 hour, the chemiluminescent signal was detected by using Super Enhanced Chemiluminescence Kit (Huaxingbio, Beijing).

### Plasmids and transfection

To construct pcDNA-Mkp1, a DNA fragment containing the rat Mkp1 coding sequences was generated by PCR amplification and cloned into HindIII and EcoRI-restricted pcDNA 3.0 (Invitrogen) as described^59^,^60^. All constructs were verified by DNA sequencing. Transient transfection of plasmids into HEK293 cells used FuGENE HD (Roch, Indianapolis, IN) transfection reagent according to the manufacturer’s instructions. After incubation for 24 h, the transfected cells were treated as indicated in figures for analysis. To get stable expression, retroviral-mediated gene transfer was performed using pMN-GFP/IRES retrovirus vector-expressing MKP-1. Infected cells were sorted by GFP signals and expanded for *in vitro* studies.

### Statistical analysis

Data were expressed as means ± SEM were statistically analyzed using one-way analysis of variance and Prism 5 for Windows (GraphPad Software, San Diego, CA). A value of *p* < 0.05 was taken to indicate statistically significant difference.

## Additional Information

**How to cite this article**: Ji, Y. *et al*. 4-Hydroxy-2-nonenal induces apoptosis by activating ERK1/2 signaling and depleting intracellular glutathione in intestinal epithelial cells. *Sci. Rep*. **6**, 32929; doi: 10.1038/srep32929 (2016).

## Supplementary Material

Supplementary Information
