# Supplementary material for: 4-Hydroxy-2-nonenal induces apoptosis by activating ERK1/2 signaling and depleting intracellular glutathione in intestinal epithelial cells
Source: Sci Rep. 2016 Sep 13;6:32929. doi: 10.1038/srep32929 (PMC5020658; doi:10.1038/srep32929)
Supplement: Supplementary Information [file srep32929-s1.pdf]

SUPPLEMENTARY INFORMATION

4-Hydroxy-2-nonenal induces apoptosis by activating ERK1/2 signalling and depleting intracellular glutathione in intestinal epithelial cells”

Yun Ji, [1] Zhaolai Dai, [1] Guoyao Wu,[1, 2] Zhenlong Wu[1], *

[1]State key Laboratory of Animal Nutrition, College of Animal Science and Technology, China Agricultural University, Beijing, 100193, P. R. China

[2]Department of Animal Science, Texas A&M University, College Station, TX 77843, USA

* To whom correspondence should be addressed:

Zhenlong Wu, Ph.D

Department of Animal Nutrition and Feed Science

College of Animal Science and Technology

China Agricultural University

Beijing, 100193, China

Tel/Fax: 86-10-62731003

Email: bio2046@hotmail.com

Supplementary Figure legends

Figure S1 (A) P53 was not implicated in ERK1/2 activation. IEC-6 cells were treated with 4-HNE as indicated for 8 h. Western blot was conducted to analyze the protein level of p53, Bax, Bcl-xL/S, pro-caspase-3, ERK1/2, and p-ERK1/2. β-actin was used as the loading control. **(B)** LC3 was not activated by 4-HNE with or without NAC pretreatment. IEC-6 cells pretreated with or without NAC (5 mM, 2 h) were treated with 4-HNE (80 μM) for the time indicated. Protein levels of LC3A/B were determined by western blot analysis. β-actin was used as the reference control.

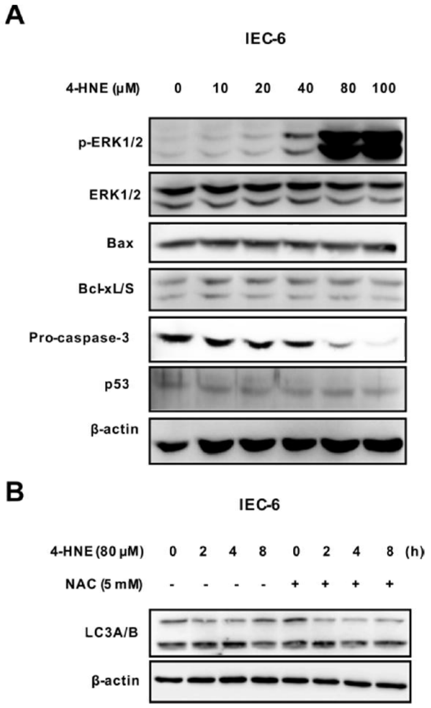

Supplementary Table S1: List of primers used for qRT- PCR analysis

Table S1A. List of primers used for qRT- PCR analysis in IEC-6 cells

Gene	Accession No.	Primers (5'-3')	Product size (bp)
GCLC	NM_012815.2	F: CATTGATTGTCGCTGGGGAG<br>R: AGATCTCCGTGTCGATGGTC	188
GSS	XM_006235308.1	F: CGTGGTGCTACTGATTGCTC<br>R: AACAGCCTTCGGTTTTGGTC	153
GSTA4	NM_001106840.1	F: TTTCAAGGCAGGGGAAGGAT<br>R: GTGTCAGTAGCATCCCGTCT	172
HO1	NM_012580.2	F: TGTAAATGCAGTGTTGGCCC<br>R: AAGGAAGACACAGGAAGGGG	144
NQO1	NM_017000.3	F: CCTGATTGTATTGGCCCACG<br>R: AGATTCGACCACCTCCCATC	103
MKP1	NM_053769.3	F: CAAAGCCCCATCACAACCTC<br>R: GAACTCAGTGGAACTCGGGA	187
β-actin	NM_031144.3	F: TGTGTTGTCCCTGTATGCCT<br>R: CCCTCATAGATGGGCACAGT	90

GCLC, glutamate cysteine ligase catalytic subunit; GSS, glutathione synthetase; GSTA4, glutathione S-transferase alpha 4; HO1, heme oxygenase1; NQO1, NAD(P)H : quinone oxidoreductase; MKP1, mitogen-activated protein kinase phosphatase-1.

Table S1B. List of primers used for qRT- PCR analysis in IPEC-1 cells

Gene	Accession No.	Primers (5'-3')	Product size (bp)
GCLC	XM_003128335.4	F: GCGGAAGTAAAATCGACGCT<br>R: TGTGAGTCCTGGGTCAATCC	89
GSS	NM_001244625.1	F: GCAGGGAAAGACACTTGTGG<br>R: ACAGGGTATGGGTTGTCGAG	120
GSTA4	XM_005666463.1	F: CGCAGGAGTCGAGTTTGATG<br>R: AGAGATGGTGCTTGTCTGCT	170
NQO1	NM_001159613.1	F: CTGGTTTGAACGTGTGCTCA<br>R: GCAGAGAGTACATGGAGCCA	131
HO1	NM_001004027.1	F: GGCCAGGTCCTCAAGAAGAT<br>R: GAAAGTGAAGAAGGCCAGGC	78
MKP1	NM_001256075.1	F: CCTTCCCCTGAGTACTAGCG<br>R: GGGGATGCTCTTGTACTGGT	235
β-actin	XM_003357928.2	F: TCTTCCAGCCCTCCTTCTTG<br>R: TCCTTCCTGATGTCCACGTC	94

GCLC, glutamate cysteine ligase catalytic subunit; GSS, glutathione synthetase; GSTA4, glutathione S-transferase alpha 4; NQO1, NAD(P)H : quinone oxidoreductase; HO1, heme oxygenase1; MKP1, mitogen-activated protein kinase phosphatase-1.
